# Continuous monitoring of cerebral blood flow during general anaesthesia in infants^☆^

**DOI:** 10.1016/j.bjao.2023.100144

**Published:** 2023-05-16

**Authors:** Sigrid D. Vik, Hans Torp, Anders H. Jarmund, Gabriel Kiss, Turid Follestad, Ragnhild Støen, Siri Ann Nyrnes

**Affiliations:** 1 Children's Clinic, St. Olavs University Hospital, Trondheim University Hospital, Trondheim, Norway; 2 Department of Circulation and Medical Imaging, Norwegian University of Science and Technology (NTNU), Trondheim, Norway; 3 Department of Computer Science, Norwegian University of Science and Technology (NTNU), Trondheim, Norway; 4 Clinical Research Unit Central Norway, St. Olavs Hospital, Trondheim, Norway; 5 Department of Clinical and Molecular Medicine, Norwegian University of Science and Technology (NTNU), Trondheim, Norway

**Keywords:** brain injury, cerebral perfusion, general anaesthesia, infants, multimodal monitoring

## Abstract

**Background:**

General anaesthesia is associated with neurocognitive deficits in infants after noncardiac surgery. Disturbances in cerebral perfusion as a result of systemic hypotension and impaired autoregulation may be a potential cause. Our aim was to study cerebral blood flow (CBF) velocity continuously during general anaesthesia in infants undergoing noncardiac surgery and compare variations in CBF velocity with simultaneously measured near-infrared spectroscopy (NIRS), blood pressure, and heart rate.

**Methods:**

NeoDoppler, a recently developed ultrasound system, was used to monitor CBF velocity via the anterior fontanelle during induction and maintenance of general anaesthesia until the start of surgery, and during recovery. NIRS, blood pressure, and heart rate were monitored simultaneously and synchronised with the NeoDoppler measurements.

**Results:**

Thirty infants, with a median postmenstrual age at surgery of 37.6 weeks (range 28.6–60.0) were included. Compared with baseline, the trend curves showed a decrease in CBF velocity during induction and maintenance of anaesthesia and returned to baseline values during recovery. End-diastolic velocity decreased in all infants during anaesthesia, on average by 59%, whereas peak systolic- and time-averaged velocities decreased by 26% and 45%, respectively. In comparison, the reduction in mean arterial pressure was only 20%. NIRS values were high and remained stable. When adjusting for mean arterial pressure, the significant decrease in end-diastolic velocity persisted, whereas there was only a small reduction in peak systolic velocity.

**Conclusions:**

Continuous monitoring of CBF velocity using NeoDoppler during anaesthesia is feasible and may provide valuable information about cerebral perfusion contributing to a more targeted haemodynamic management in anaesthetised infants.

Infants undergoing surgery are at increased risk of subsequent neurodevelopmental delay.^1^, ^2^, ^3^ One pathophysiological mechanism potentially contributing to such delay may be disturbances in cerebral perfusion during general anaesthesia.^4^^,^^5^ Preterm infants and sick neonates are particularly vulnerable to such disturbances as a result of impaired cerebral autoregulation leading to a pressure-passive cerebral circulation and reduced ability to maintain stable cerebral perfusion during general anaesthesia.^6^, ^7^, ^8^, ^9^

The most common clinical practice to ensure haemodynamic stability during general anaesthesia is to optimise systemic circulation with the overall goal to optimise end-organ perfusion, including the brain.^5^^,^^10^^,^^11^ However, the definition of systemic hypotension in infants is unclear,^6^^,^^12^, ^13^, ^14^, ^15^ and there are individual differences in the upper and lower limit of intact cerebral autoregulation in sick infants.^7^^,^^8^^,^^10^ Furthermore, the impact of anaesthetic drugs and the impact of changes in physiological variables make the assessment of cerebral perfusion challenging.^16^, ^17^, ^18^, ^19^ The complexity and challenges in the assessment of cerebral perfusion require better monitoring tools that can provide decision support and a more individualised assessment of cerebral perfusion.

Doppler measurements have been used to assess cerebral haemodynamics since the introduction of transcranial Doppler in 1982.^20^ However, continuous measurements with transcranial Doppler require a transducer coupled to a large, rigid head accessory designed for adults, and a trained operator to locate the vessel of interest. Although infants have the perfect acoustic window through the anterior fontanelle, continuous monitoring of cerebral perfusion assessed with Doppler measurements has not yet been possible. Such monitoring might identify individuals with reduced cerebral perfusion.

We hypothesised that continuous monitoring of cerebral blood flow (CBF) velocity to assess cerebral perfusion during general anaesthesia might reveal new insights into cerebral haemodynamics. The primary aim was to use NeoDoppler,^21^ a new ultrasound system for continuous monitoring, to evaluate CBF velocity during general anaesthesia in infants undergoing noncardiac surgery. Secondary aims were to explore associations of CBF velocity with simultaneously measured near-infrared spectroscopy (NIRS), blood pressure, and heart rate.

## Methods

### Design and study population

This observational study included a convenience sample of consecutively recruited infants with an open fontanelle undergoing noncardiac surgery at the Department of Paediatric surgery, St. Olavs Hospital, Trondheim University Hospital Norway, between May 2020 and May 2022. CBF velocity was continuously recorded before, during induction and maintenance of anaesthesia until the start of surgery, and during recovery, using NeoDoppler. This is an operator-independent, noninvasive ultrasound system providing continuous non-angle-corrected velocity measurements from vessels at different depths of the brain simultaneously.^21^ The velocity measurements included peak systolic velocity, time-averaged maximum velocity, and end-diastolic velocity. Based upon these measurements, pulsatility index (=[peak systolic velocity–end-diastolic velocity]/time-averaged maximum velocity) and resistive index (=[peak systolic velocity–end-diastolic velocity]/peak systolic velocity) were continuously calculated and displayed.^21^ As indicators of cerebral haemodynamics, peak systolic velocity is considered to mainly reflect systemic factors, such as cardiac output and systolic blood pressure, whereas end-diastolic velocity is the most sensitive marker to changes in cerebral haemodynamics, such as changes in peripheral resistance and diastolic blood pressure.^22^ Time-averaged maximum velocity is considered to be directly related to cerebral perfusion,^23^ and an increase in time-averaged maximum velocity may be explained by increased blood pressure or reduced peripheral resistance. NIRS was used to measure regional cerebral oxygenation. Before anaesthesia, during maintenance of anaesthesia, and during recovery heart rate, oxygen saturation and blood pressure were obtained. All variables were recorded and synchronised in real time or, in some cases, vital signs were recorded every 30 s and later synchronised with the velocity measurements and NIRS. Baseline blood pressure was measured noninvasively before induction in a calm infant. During induction, blood pressure was not measured, whereas during maintenance of anaesthesia blood pressure was measured either continuously (invasively) or noninvasively every 5 min. Interventions for blood pressure management were performed at the discretion of the attending anaesthesiologist.

### NeoDoppler

NeoDoppler consists of a small, lightweight ultrasound probe, a scanner, and a user interface with display. The probe operates at a frequency of 7.8 MHz during monitoring. The transmitted beam consists of plane waves and covers a cylindrically shaped area with a diameter of 1 cm and measures velocities to a depth of 35 mm. The clutter filter was set to 60 Hz. The Doppler signal is guided by the M-mode image and no morphological details are obtained.^21^ The NeoDoppler probe was attached over the anterior fontanelle of the infant's head with a specifically designed soft hat. The NeoDoppler probe and the velocity measurements were continuously surveyed and optimised during monitoring by the first author.

### Other measurements

Details of the infant's clinical diagnosis, gestational age, postnatal age at surgery, birth weight, and weight at surgery were collected from the medical records.

Details of anaesthetic management, including type of medication, drug dosage, and time, and airway management and fluid management during induction and maintenance of anaesthesia were registered. If the infant's trachea was to be intubated in the operating room, the standard induction was i.v. propofol, fentanyl, and rocuronium. During maintenance of anaesthesia, sevoflurane in combination with fentanyl and rocuronium were standard. Events during anaesthesia were documented. The management and interventions during anaesthesia were at the discretion of the attending anaesthesiologist, who was blinded to the NeoDoppler measurements.

### Statistical methods

Real-time data acquisition of CBF velocity and post processing were done with in-house software developed in MATLAB (MathWorks® R2021a, Natick, MA, USA). Statistical analyses were performed using SPSS Statistics version 27.0 (IBM, Armonk, NY, USA) except for the linear mixed models which were performed in R (version 4.2.1, R Foundation for Statistical Computing, Vienna, Austria) with the lme4 package.^24^ Trend curves based on the continuous recording of CBF velocity variables, NIRS, heart rate, and blood pressure were obtained and inspected for each infant. We analysed those parts of the recordings when the curves suggested stable CBF velocity. In order to select these periods as objectively as possible, we analysed CBF velocity during 200 heart beats in the awake infant before induction as baseline (P1); during the first stable period after tracheal intubation when blood pressure measurements were established (P3: ‘early anaesthesia’), immediately before surgery (P4: ‘late anaesthesia’), immediately after the end of surgery (P5: ‘early recovery’), and the last observed period during recovery (P6: ‘late recovery’). During induction of anaesthesia, CBF velocities during the last 10 heartbeats just before tracheal intubation were analysed (P2). To compare the different periods, we used the fraction of the baseline measurement for all variables. However, since blood pressure at baseline was mainly acquired noninvasively, we also calculated the blood pressure during anaesthesia as a fraction of invasive blood pressure measurements during recovery.

Linear mixed models were used to study phase-to-phase changes in CBF velocity and how changes in CBF velocity were impacted by corresponding changes in blood pressure. By using linear mixed models, we were able to include infants whose measurements were missing at some time points. The CBF velocity variables peak systolic velocity, time-averaged maximum velocity, and end-diastolic velocity were averaged for each participant for each period (P1–P6) and used as dependent variables in three separate models. The distributions of the CBF velocity variables were explored with QQ plots and were found to depart from the normal distribution. Thus, log-transformation of these variables was performed to obtain better approximations to the normal distribution. The different time points (P1–P6) were included as categorical covariates and a random intercept for participant was included to account for within-subject correlations. Bootstrapping was used to establish 95% confidence intervals by resampling participants at random, with replacement, and constructing new linear mixed models for each bootstrap sample.^25^ This was repeated 1000 times and the 2.5 and 97.5 percentiles for each coefficient were used as lower and upper limits, respectively, of the corresponding confidence interval. The procedure was repeated with mean arterial blood pressure (MAP) as a separate covariate to see whether changes in blood pressure could explain the changes in CBF velocity. In addition, sex, gestational age, birth weight, surgery before or after 7 days of age, diagnosis, and blood pressure were explored separately as additional covariates.

We defined hypotension as an MAP <35 mm Hg,^26^^,^^27^ and used Mann–Whitney *U*-tests to compare CBF velocity between those with and without hypotension.

### Safety

The attending anaesthesiologist was free to remove the NeoDoppler probe if the probe was interfering with the clinical management of the infant. The settings that influence mechanical and thermal index were set in advance. Because of the unfocused probe, the highest thermal index is at the skin surface and the temperature decreases significantly in the depths of the brain.^21^ After removing the probe, the skin was inspected for possible erythema or damage, or pressure marks.

The Regional Committee for Medical and Health Research Ethics in Central Norway, the Norwegian Directorate of Health, and The Norwegian Medicines Agency approved the study. Informed, written parental consent was obtained by a research nurse or the first author.

## Results

### Patient data

In total, 30 infants (20 males) were included. Based on the main surgical diagnosis we defined four groups; atresia of the gastrointestinal tract (*n*=10), abdominal wall defects (*n*=6), other major surgery (*n*=8) (e.g. congenital diaphragmatic hernia, patent ductus arteriosus ligation), and minor surgery (*n*=6) (e.g. inguinal hernia, venous access port). Background variables are shown in Table 1.

**Table 1:** Background variables.

	*N* (%)	Median (range min–max)
Total	30 (100)	
Sex		
Male	20 (66.7)	
Gestational age (weeks)		
All infants	30 (100)	35.65 (24.6–41.4)
<32 weeks	7 (23.3)	
32–36 weeks	11 (36.7)	
≥37 weeks	12 (40.0)	
Birth weight (g)		
All infants	30 (100)	2335.0 (591–5920)
<1500 g	7 (23.3)	
1500–2500 g	9 (30.0)	
≥2500 g	14 (46.7)	
Postmenstrual age (weeks) at surgery		
All infants	30 (100)	37.55 (28.6–60.0)
Age at surgery (days)		
All infants	30 (100)	2.02 (0.10–140)
<7 days	20 (66.7)	
≥7 days	10 (33.3)	
Weight at surgery (g)		
All infants	30 (100)	2587.5 (930–9530)

### Procedures

I.V. induction was used in 24 of the 25 infants whose tracheas were intubated in the operating room. Gas induction with sevoflurane 4% followed by fentanyl and rocuronium was used in one infant. The lungs of four infants were being mechanically ventilated before entering the operating room, and the airway of one infant was managed using a laryngeal mask airway during anaesthesia. Inhalation maintenance of anaesthesia was used in 28 infants in combination with fentanyl and rocuronium. Five infants had additional regional anaesthesia. Invasive blood pressure during anaesthesia was used in 21 infants. For all except the six infants with minor surgery, mechanical ventilation of the lungs was continued during recovery. Further information regarding anaesthetic management is shown in Table 2.

**Table 2:** Anaesthetic management of the infants monitored with NeoDoppler. *P*co_2_, partial pressure of carbon dioxide.

	N	Median (range min–max)
Propofol during induction (mg kg^−1^)	25	3.88 (2.67–5.29)
Fentanyl during induction (μg kg^−1^)	23	1.82 (0.52–3.62)
Rocuronium during induction (mg kg^−1^)	28	0.79 (0.25–1.98)
Time from intubation to start of surgery (min)	21	76.60 (15.32–134.4)
Sevoflurane % during maintenance of anaesthesia	24	1.6 (0.85–2.55)
Fentanyl during maintenance of anaesthesia (μg kg^−1^)	27	1.98 (0.52–7.53)
Rocuronium during maintenance of anaesthesia (mg kg^−1^)	20	0.58 (0.29–6.45)
Inspired oxygen during induction of anaesthesia (%)	21	98.4 (95.3–101.1)
Inspired oxygen during maintenance of anaesthesia (%)	24	48.65 (28.5–79.7)
Inspired oxygen during recovery (%)	19	24.1 (21–30)
*P*co_2_ (capillary) before anaesthesia (kPa)	23	6.3 (4.98–10.2)
End tidal CO_2_ during maintenance of anaesthesia (kPa)	24	4.46 (3.22–5.85)
*P*co_2_ (capillary) during recovery (kPa)	21	5.8 (4.32–7.9)

Adjustment of the probe during monitoring was sometimes necessary to optimise the Doppler signal; this sometimes caused small changes in the beam-to-flow angle leading to corresponding changes in the velocity measurements. Nine infants did not have complete recordings obtained from the same vessel from baseline (P1) to late recovery (P6), leaving 21 infants with continuous recordings during all time periods (Supplementary Fig. S1). The main reason for missing time periods was the need for probe adjustment (Supplementary Table S1). Of the 21 infants with complete recordings during anaesthesia and recovery (P1–P6), three were supported with mechanical ventilation at baseline.

The median duration of monitoring with NeoDoppler was 5 h 10 min (range 1 h 56 min to 10 h 28 min).

### Trend curves

Variability in CBF velocity was first studied by observing the individual trend curves. Figure 1 shows an example of a typical trend curve where changes in CBF velocity, blood pressure, arterial saturation, NIRS, and heart rate can be followed from baseline (P1) until late recovery (P6). Figure 2 shows changes in the Doppler waveforms before, during, and after anaesthesia.

**Fig. 1:**
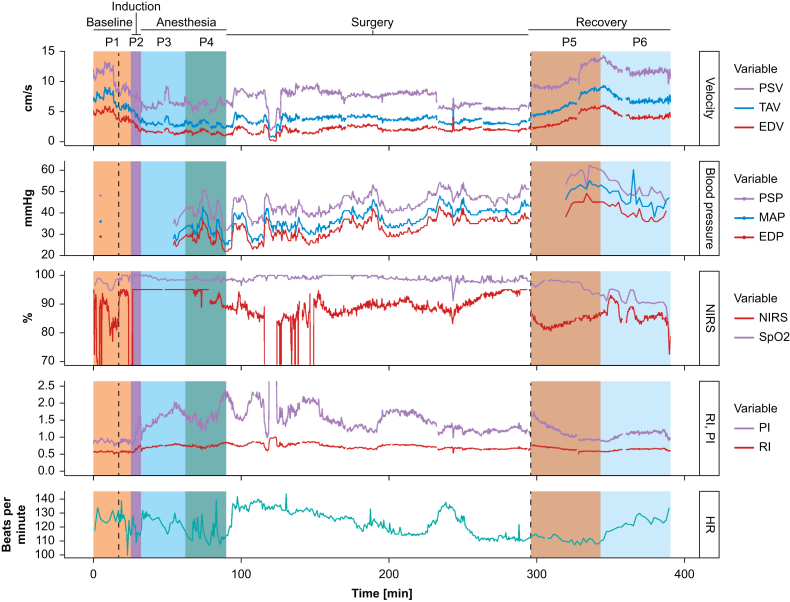
Continuous trend curves displaying multimodal monitoring during anaesthesia. The figure shows characteristic patterns of cerebral blood flow velocity (CBF velocity) and blood pressure, near-infrared spectroscopy (NIRS) and heart rate in one infant with gastrointestinal atresia, gestational age 37 weeks. There is a decrease in CBF velocity during induction (P2). After intubation (P3), the CBF velocity stabilises at a level below baseline and remains stable until surgery commences (P4). During this period, from P3 to P4, NIRS is stable at high values (95%), whereas invasive mean arterial pressure (MAP) measurements vary between 25 and 43 mm Hg. CBF velocities return to baseline values during recovery, whereas blood pressure stabilises at a higher level than baseline. During recovery, NIRS is still high, but lower than during anaesthesia (P3–P4), and comparable with baseline values. NIRS sensor for infants and neonates, INVOS™ 5100c OxyAlert™ (Medtronic Parkway, Minneapolis, MN, USA). Synchronising of the monitoring variables was done using pyMIND (https://pymind.readthedocs.io/en/latest/), an open-source Python-based software designed to acquire and integrate multi-modal scientific data from medical devices. The software was modified in-house and extended to capture NIRS data in addition to invasive measurements (e.g. Philips IntelliVue). On the receiver side a common time axis was maintained to which all measurements were synchronised. The highest available temporal resolution was captured for all signals and all data were saved into HDF format for offline processing. EDP, end-diastolic pressure; EDV, end-diastolic velocity; HR, heart rate; MAP, mean arterial pressure; NIRS, near-infrared spectroscopy; P1, baseline; P2, induction of anaesthesia; P3, early anaesthesia; P4, late anaesthesia; P5, early recovery; P6, late recovery; PI, pulsatility index; PSP, peak systolic pressure; PSV, peak systolic velocity; RI, resistive index; SpO_2_, arterial oxygenation; TAV, time-averaged maximum velocity.

**Fig. 2:**
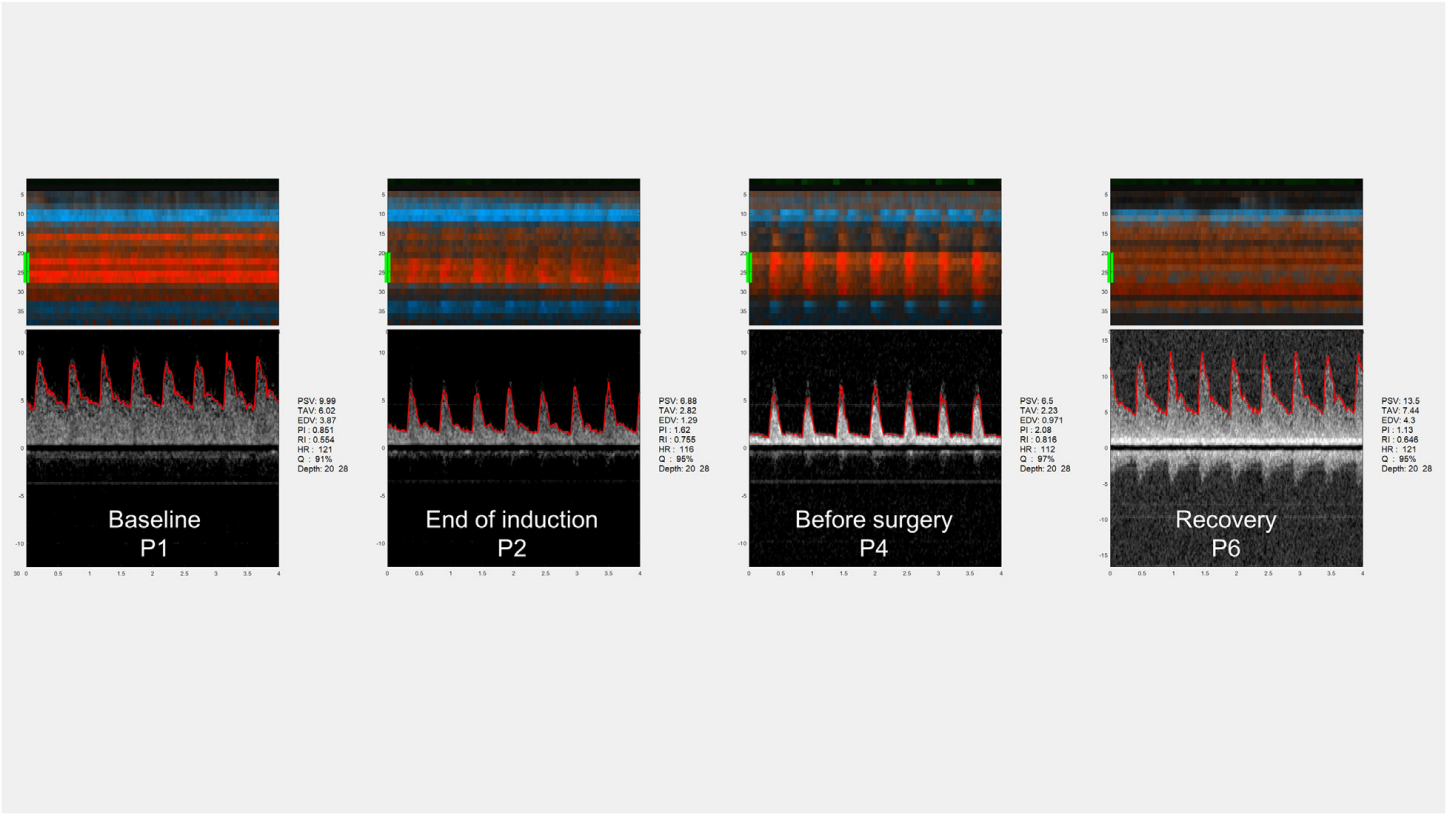
Changes in Doppler waveforms before, during and after anaesthesia. The figure shows representative examples of Doppler waveforms from the same infant as Figure 1 during the different periods. Detailed morphology of the brain by a two-dimensional greyscale image is not obtained, instead the depth-versus-time colour M-mode is used to place the sample volume where there is a strong arterial signal. The colour M-mode in the upper panel shows that the measurements are obtained in the same depth, 20–28 mm, during the different periods. The time scale is in seconds. The Doppler signals suggest a decrease in velocities during anaesthesia compared with baseline and return to baseline velocities during recovery. EDV, end-diastolic velocity; HR, heart rate; PI, pulsatility index; PSV, peak systolic velocity; Q, quality; RI, resistive index; TAV, time-averaged maximum velocity.

**Fig. 3:**
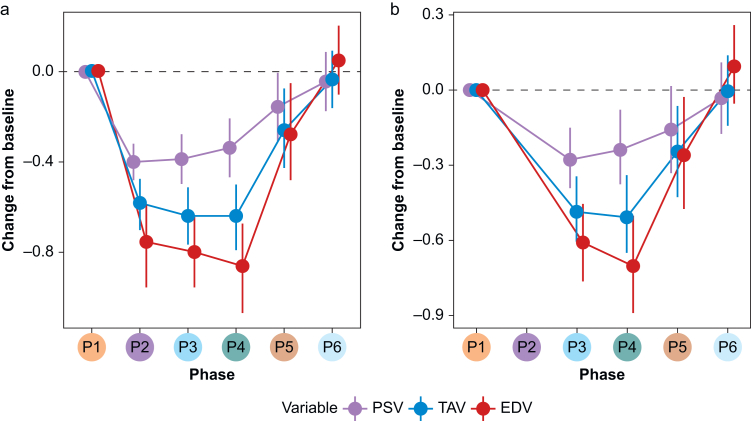
Changes of the cerebral blood flow velocity from baseline to late recovery. Changes from baseline are shown as natural log transformed values, including 95% confidence interval, and show a decrease in cerebral blood flow velocity unadjusted (a) and adjusted for mean arterial blood pressure (b). Data from *n*=27. EDV, end-diastolic velocity; P1, baseline; P2, induction of anaesthesia; P3, early anaesthesia; P4, late anaesthesia; P5, early recovery; P6, late recovery; PSV, peak systolic velocity; TAV, time-averaged maximum velocity.

Anaesthesia was associated with a statistically significant decrease in peak systolic velocity, time-averaged maximum velocity, and end-diastolic velocity; the largest decrease was in end-diastolic velocity (Fig 3a; Table 3). There was no evidence that sex, gestational age, birth weight, surgery before or after 7 days of age or diagnosis were associated with the CBF velocity variables (data not shown). The decreases in the CBF velocity variables persisted when we adjusted for MAP (Fig 3b).

**Table 3:** Absolute values of cerebral blood flow velocities, heart rate, NIRS, and blood pressure measured at baseline (P1), during maintenance of anaesthesia (P2–P4) and during recovery (P5–P6), and fraction of baseline. ∗*P*-values from linear mixed model, log-transformed scale. ^†^Mean fraction of baseline at P2–P6, 21 infants at P1, P3–P6, 18 infants at P2. ^‡^*N*=26. ^¶^*N*=27. ^§^*N*=25. ^||^*N*=22. ^#^*N*=19. ∗∗*N*=17.^††^*N*=20. EDP, end-diastolic pressure; EDV, end-diastolic velocity; HR, heart rate; IQR, inter-quartile range; MAP, mean arterial pressure; NIRS, near-infrared spectroscopy; PI, pulsatility index; PSP, peak blood pressure; PSV, peak systolic velocity; RI, resistive index; TAV, time-averaged maximum velocity.

		Baseline (P1)	Induction (P2)		During anaesthesia				During recovery			
					Early (P3)		Late (P4)		Early (P5)		Late (P6)	
		*N*=25	*N*=22	*P*-values∗	*N*=28	*P*-values∗	*N*=27	*P*-values∗	*N*=25	P-values∗	*N*=25	*P*-values∗
PSV	Median (IQR)	9.82 (7.62–18.00)	7.23 (4.69–10.03)	<0.001	6.76 (5.08–15.99)	<0.001	7.74 (5.5–14.28)	<0.001	9.91 (7.58–13.48)	0.024	11.26 (7.60–14.98)	0.487
	Fraction^†^	1	0.67 (0.39–1.0)		0.69 (0.38–1.04)		0.74 (0.42–1.19)		0.92 (0.31–1.88)		1.01 (0.57–1.82)	
TAV	Median (IQR)	6.33 (5.22–9.69)	3.55 (2.61–4.67)	<0.001	3.17 (2.51–6.85)	<0.001	3.51 (2.84–6.26)	<0.001	6.0 (3.76–8.77)	0.001	7.40 (4.66–8.94)	0.637
	Fraction^†^	1	0.57 (0.30–1.03)		0.54 (0.26–0.81)		0.55 (0.25–1.0)		0.86 (0.29–2.26)		1.04 (0.52–1.95)	
EDV	Median (IQR)	3.46 (2.71–4.63)	1.57 (1.00–2.46)	<0.001	1.49 (1.22–2.55)	<0.001	1.66 (1.17–2.04)	<0.001	3.17 (1.54–4.59)	0.01	4.27 (2.77–5.05)	0.671
	Fraction^†^	1	0.51 (0.16–1.06)		0.47 (0.20–0.77)		0.41 (0.13–0.90)		0.88 (0.30–2.70)		1.16 (0.44–2.09)	
PI	Median (IQR)	1.04 (0.87–1.28)	1.49 (1.19–1.87)	<0.001	1.53 (1.34–1.98)	<0.001	1.68 (1.44–2.11)	<0.001	1.23 (1.05–1.60)	0.009	0.97 (0.98–1.20)	0.482
	Fraction^†^	1	1.40 (0.85–2.17)		1.49 (0.94–2.17)		1.68 (0.99–2.53)		1.20 (0.6–1.92)		0.95 (0.53–1.29)	
RI	Median (IQR)	0.65 (0.58–0.74)	0.75 (0.67–0.83)	<0.001	0.76 (0.70–0.83)	<0.001	0.78 (0.73–0.85)	<0.001	0.71 (0.65–0.79)	0.037	0.63 (0.59–0.71)	0.345
	Fraction^†^	1	1.13 (0.89–1.52)		1.15 (0.97–1.38)		1.20 (0.97–1.58)		1.06 (0.72–1.46)		0.96 (0.76–1.12)	
HR	Median (IQR)	139.59 (128.74–155.19)	129.54 (121.68–138.44)	0.351	137.35 (128.04–151.50)	0.196	125.88 (116.77–141.08)	0.027	127.11 (118.92–146.83)	0.534	133.48 (122.84–152.31)	0.732
	Fraction^†^	1	0.98 (0.81–1.25)		1.04 (0.80–1.36)		0.97 (0.70–1.27)		1.00 (0.76–1.27)		1.02 (0.83–1.24)	
NIRS	Median (IQR)	88.68 (72.69–93.93)	90.87 (82.18–95)	0.064	83.05 (75.25–95.0)	0.118	85.88 (75.74–95.0)^§^	0.424	78.35 (67.93–85.20)	0.085	82.11 (73.13–87.21)	0.316
	Fraction^†^	1	1.09 (0.85–1.31)∗∗		1.06 (0.91–1.20)^a^		1.02 (0.79–1.18)^#^		0.96 (0.80–1.13)^a^		0.97 (0.53–1.25)^a^	
PSP	Median (IQR)	63.75 (56.0–79.67)^‡^	—	—	47.18 (43.00–56.87)^¶^		47.50 (43.70–53.97)^‡^		62.68 (50.18–71.85)^||^		57.39 (46.93–67.57)^||^	
	Fraction^†^	1^a^	—	—	0.75 (0.45–1.06)^#^		0.75 (0.51–1.03)^#^		0.98 (0.61–2.01)∗∗		0.90 (0.62–1.20)∗∗	
MAP	Median (IQR)	47.33 (37.13–55.92)^‡^	—	—	38.68 (34.00–40.73)^¶^		36.49 (32.04–42.00)^‡^		50.74 (35.75–59.57)^||^		44.33 (38.77–56.93)^||^	
	Fraction^†^	1^††^	—	—	0.80 (0.46–1.20)^#^		0.80 (0.43–1.12)^#^		1.13 (0.66–2.84)∗∗		1.03 (0.76–1.54)∗∗	
EDP	Median (IQR)	36.83 (29.63–47.08)^‡^	—	—	31.2 (25.51–33.00)^¶^		28.49 (24.75–32.84)^‡^		45.05 (26.00–49.69)^||^		37.57 (31.42–48.71)^||^	
	Fraction^†^	1^a^	—	—	0.81 (0.39–1.26)^#^		0.79 (0.36–1.12)^#^		1.26 (0.6–3.20)∗∗		1.19 (0.73–2.49)∗∗	

The variation in absolute values of the velocity variables were large (Table 3). The fractions of baseline values of the velocity variables during anaesthesia were analysed in the 21 infants with complete velocity measurements. Of these, 20 infants had blood pressure measurements at baseline, 19 during maintenance, and 17 infants during recovery (Table 3). Compared with baseline, NIRS did not change during anaesthesia, whereas MAP decreased on average by 20%, mean end-diastolic velocity by 59%, mean peak systolic velocity by 26%, and pulsatility index increased by 68% (Table 3). Mean end-diastolic velocity was the only variable that decreased in all infants (Table 3). Restricted to infants with invasively measured blood pressure the MAP during maintenance was reduced by 23% compared with recovery. All the variables returned to baseline values during late recovery (Table 3, Fig 3a).

Just before surgery (P4), infants with an MAP <35 mm Hg (*N*=12) had significantly lower end-diastolic velocity (median: 1.3; inter-quartile range [IQR] 0.84–1.63) than infants with an MAP ≥35 mm Hg (*n*=14) (median: 1.8; IQR 1.48–3.34; *P*=0.011).

### Safety

No infant needed the NeoDoppler probe to be removed during the procedure. Mechanical and thermal indices were 0.06 and 0.57, respectively, during all recordings. There were no visible skin marks after removing the NeoDoppler probe after several hours of monitoring.

## Discussion

This is the first study to use NeoDoppler in multimodal monitoring to assess cerebral perfusion during anaesthesia in infants undergoing noncardiac surgery. We have shown that continuous monitoring with NeoDoppler is feasible and provides additional information on cerebral perfusion not obtained with standard monitoring. There were significant changes in all CBF velocity variables during anaesthesia, particularly in end-diastolic velocity which was reduced by 59% compared with baseline. The reduction in end-diastolic velocity was greater in hypotensive compared with normotensive infants.

Doppler measurements to assess cerebral perfusion during anaesthesia and surgery in infants with an open fontanelle have been reported in previous studies.^28^, ^29^, ^30^, ^31^ However, these studies used intermittent measurements at predefined time points and were dependent on trained personnel. In contrast, NeoDoppler enables continuous, operator-independent measurements which may add unique pathophysiological knowledge. The reduction in end-diastolic velocity in our patients may indicate reduced cerebral perfusion as end-diastolic velocity is the most sensitive marker of haemodynamic changes.^22^^,^^26^ The same pattern is also seen in other pathological conditions, such as haemodynamically significant patent ductus arteriosus, asphyxia, and sepsis.^22^ By contrast, the reduction in end-diastolic velocity may also indicate increased peripheral resistance.^30^^,^^31^ However, the less marked reduction in peak systolic velocity and the significant decrease in time-averaged maximum velocity are consistent with our interpretation that the observed decrease in end-diastolic velocity indicates reduced CBF.

The decrease in end-diastolic velocity in our patients was independent of gestational age, birth weight, age at surgery, and diagnosis. This means that despite a heterogenous group of infants, the observed changes in CBF velocity during anaesthesia are robust. When adjusting for MAP, there was only a marginal reduction in peak systolic velocity, whereas the decrease in end-diastolic velocity persisted. We speculate that these results indicate that some infants had a suboptimal blood pressure during anaesthesia which, in combination with impaired cerebral autoregulation, resulted in reduced end-diastolic velocity. Whether a significant reduction in end-diastolic velocity may be related to an increased risk of adverse neurodevelopment outcome warrants further studies.

The blood pressure management during anaesthesia in our study was consistent with current recommendations.^6^^,^^11^^,^^26^ In clinical practice, the anaesthesiologist relies on blood pressure along with their overall clinical assessment.^10^^,^^14^^,^^27^ However, the optimal blood pressure in infants during anaesthesia is unclear,^14^^,^^32^^,^^33^ and a reliable method to monitor brain perfusion directly, which would guide blood pressure management, is lacking. Consistent with Rhondali and colleagues,^26^ end-diastolic velocity in our study was lower in infants with hypotension compared with those who were normotensive. However, the results of this study do not allow general recommendations regarding lower limits of MAP during anaesthesia. With NeoDoppler, a more individual assessment of cerebral perfusion is possible and may in future contribute to more targeted blood pressure control during anaesthesia.

The infants in our study maintained high and stable NIRS values during procedures. This may suggest adequate cerebral perfusion,^34^ but it could also be an effect of increased oxygen supply^9^^,^^32^^,^^34^ or reduced cerebral metabolism with reduced consumption of oxygen.^34^ These uncertainties in the interpretation of NIRS and the lack of evidence of improved neurological outcome when monitoring with NIRS, may explain why NIRS monitoring is seldom used in infants undergoing noncardiac surgery. Further studies are needed to clarify whether NIRS or CBF velocity are the most appropriate measures of cerebral perfusion, or if they should be used as complementary measures.

### Weaknesses and strengths

One limitation with NeoDoppler is that in contrast to conventional ultrasound examination, we do not know exactly which vessels are being measured. This leads to a variability of the absolute measurements such that comparison of absolute values between patients, and across studies, is difficult. However, the main advantage of NeoDoppler is that it is operator-independent and enables continuous monitoring of CBF velocity during long time periods. The large variability in the absolute measurements is a strong argument for measuring trends instead of intermittent measurements. Thus, it is possible to monitor and assess cerebral perfusion directly, noninvasively, and at the operating table. This is a unique and original feature of NeoDoppler.^21^ In this research set-up, the Doppler measurements were continuously surveyed by the first author to note events and anaesthetic management, performing small adjustments of the probe to optimise the signal if necessary. In clinical use, NeoDoppler is intended to be part of the regular monitoring system with an alert to indicate poor Doppler signals requiring adjustments of the probe.

A major controversy when using Doppler measurements to assess cerebral perfusion is the extent that velocity measurements reflect CBF. The velocity measurements are dependent on the diameter of the vessel, which is continuously changing. However, several studies have shown good correlation between velocity measurements and CBF, especially when the changes in velocity can be followed continuously, which is the case with transcranial Doppler.^35^, ^36^, ^37^, ^38^, ^39^, ^40^ The typical trend curve obtained with NeoDoppler shows changes in velocity measurements compared with baseline. These changes in velocity further support that continuous velocity measurements may reflect CBF.

A majority of the infants in this study had only noninvasive blood pressure measurements at baseline. However, when we restricted the analyses to infants with invasively measured blood pressure and compared the blood pressure values during anaesthesia with measurements at recovery, the results were essentially unchanged.

The strength of this study was that the infants were monitored under stable conditions, underwent similar anaesthetic management without any significant haemorrhage, and the same investigator was present during the full monitoring period for all patients. This enabled simultaneous and reliable measurements of the three variables—blood pressure, NIRS, and CBF velocity—which, when seen in relation to each other, may contribute to a more comprehensive and individualised understanding and assessment of CBF and provide new possibilities to optimise brain perfusion during anaesthesia.

## Conclusions

Continuous monitoring of CBF velocity with NeoDoppler revealed a significant decrease in end-diastolic velocity during anaesthesia in infants with an open fontanelle undergoing noncardiac surgery. Our results suggest that continuous monitoring of CBF velocity with NeoDoppler during anaesthesia is feasible and may provide valuable information to improve haemodynamic management. Whether improved CBF velocity may reduce the risk for neurodevelopmental impairments in infants undergoing noncardiac surgery should be the aim for future research.

## Authors’ contributions

Collected and compiled the data, did the data analyses, wrote the first draft, and completed the manuscript: SDV.

Inventor of the technology: HT.

Supervised the collection of the data: HT, GK, SAN.

Supervised post-processing of the data: HT, GK.

Contributed to finalising the manuscript: HT, GK, AJH, TF, RS, SAN.

Enabled synchronising of the data from Philips monitor with the velocity parameters: GK.

Contributed to data analyses, interpretation and presentation of the data: AJH.

Proposed and supervised the statistical methods and the data analyses: TF.

Participated in the interpretation of the data: TF, RS, SAN.

Contributed to the design of the study: RS, SAN.

Contributed to drafting the manuscript: SAN.

## Acknowledgements

We would like to thank the infants who participated in this study and their parents, and the nurses and doctors in the neonatal intensive care unit and in the operating room for the implementation of this study. Neonatal intensive care unit nurse, Wenche Skånøy, obtained consent from the parents. Thanks to Associate Professor Emerita Nancy Eik-Nes for proofreading the English language of this article. Professor and paediatric anaesthesiologist, Eirik Skogvoll, has read and provided valuable input to the manuscript.

## Declarations of interest

NTNU and St. Olavs Hospital, Trondheim University Hospital, may benefit financially from a commercialisation of the ultrasound equipment through future possible intellectual properties; this may include financial benefits to authors of this article. SDV, HT, RS, and SAN are co-inventors of NeoDoppler. HT and SAN have part time positions in CIMON Medical, the company that is responsible for commercialisation of NeoDoppler. HT and SAN are among the shareholders and SAN is board member in CIMON Medical. AHJ, GK, and TF declare that they have no conflicts of interest.

## Funding

PhD grant from the Faculty of Medicine and Health Science, Norwegian University of Science and Technology (NTNU) (Reference 81115200) to SDV. Joint Research Committee between St. Olavs Hospital and the Faculty of Medicine and Health Sciences, 10.13039/100009123NTNU (Reference 2019/38881 and 2018/42794, respectively) to AHJ and SAN. The 10.13039/501100005416Research Council of Norway in the initial phase.

## Figures and Tables

**Figs1:**
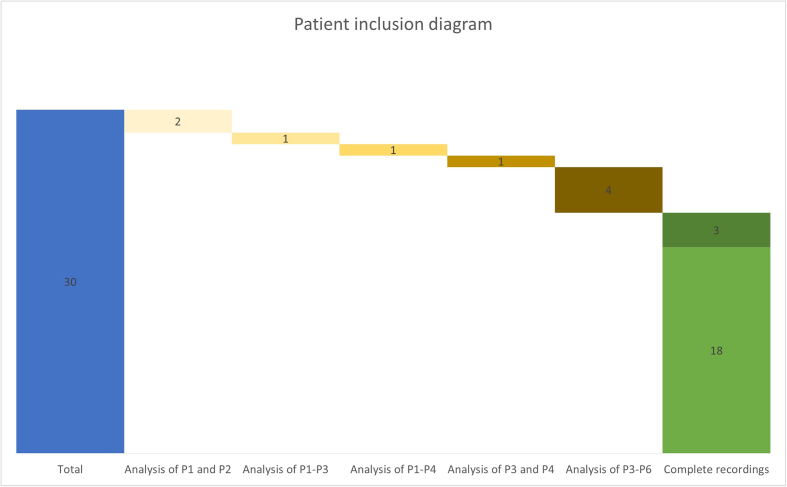
Patient inclusion diagram.
