# Supplementary material for: Continuous monitoring of cerebral blood flow during general anaesthesia in infants
Source: BJA Open. 2023 May 16;6:100144. doi: 10.1016/j.bjao.2023.100144 (PMC10430850; doi:10.1016/j.bjao.2023.100144)
Supplement: Multimedia component 1 [file mmc1.docx]

**Supplementary Table 2: Reasons for incomplete recordings in nine infants**

| **Reasons** | **N of infants** | **Type of surgery** | **Gestational age (weeks)** | **Measurements obtained** | **Measurements used** |
| --- | --- | --- | --- | --- | --- |
| Probe adjustment | 1 | Minor surgery | 30.0 | P1-P6 | P1 -P2 |
| Probe adjustment | 1 | Gastrointestinal atresia | 41.1 | P1-P4 | P1 -P2 |
| Probe removed (fixation) | 1 | Gastrointestinal atresia | 32.4 | P1-P3 | P1 -P3 |
| Long duration surgery^*^ | 1 | Gastrointestinal atresia | 36.6 | P1-P4 | P1-P4 |
| Suboptimal quality (fixation) and long duration^**^ | 1 | Other major surgery | 39.0 | P1-P4 | P3-P4 |
| Suboptimal quality (fixation) | 2 | Gastrointestinal atresia | 34.6/ 35.4 | P1-P6 | P3-P6 |
| Probe fixation after P2^***^ | 1 | Other major surgery | 39.0 | P3-P6 | P3-P6 |
| Infant intubated before admission^****^ | 1 | Abdominal wall defects | 31.1 | P3-P6 | P3-P6 |
| Total with incomplete recordings | 9 |  |  |  |  |

P1=baseline, P2=induction of anesthesia, P3=early anaesthesia, P4= late anaesthesia, P5= early recovery and P6= late recovery

*12 hours anaesthesia, **13 hours anaesthesia, ***Intravenous line on the head, which was removed after induction of anesthesia, ****Transfer from another hospital and directly entering the operating room, already intubated
